# Cutaneous *Mycobacterium shigaense* Infection in Immunocompetent Woman, China

**DOI:** 10.3201/eid1905.121022

**Published:** 2013-05

**Authors:** Pangen Cui, Varalakshmi Vissa, Wei Li, Xiaodong Zhang, Lin Lin, Hongsheng Wang, Xiaolin Liu, Qinxue Wu, Wenkai Zong

**Affiliations:** Institute of Dermatology, Chinese Academy of Medical Sciences, Jiangsu Key Laboratory of Molecular Biology for Skin Diseases and STIs, Jiangsu, China (P. Cui, X. Zhang, L. Lin, H. Wang, X. Liu, Q. Wu, W. Zong);; Colorado State University, Fort Collins, Colorado, USA (V. Vissa, W. Li)

**Keywords:** cutaneous, infection, Mycobacterium shigaense, bacteria, immunocompetent, tuberculosis and other mycobacteria, NTM, nontuberculous mycobacteria, China

**To the Editor:** *Mycobacterium shigaense* is a novel, slow-growing, scotochromogenic mycobacterium (*1*), initially reported in 2012 as an opportunistic pathogen isolated from skin biopsy samples from a patient with a history of Hodgkin disease and severe cellular immunodeficiency. We describe the identification of this species in a chronic cutaneous infection in an immunocompetent woman. 

A 56-year-old woman was admitted to our inpatient department in August 2011 with reddish papules, nodules, plaques, and scars on her face and neck (Figure, panel A) that had developed over >1 year, starting in June 2010. Initially, a few small papules appeared on her face; the primary papules gradually enlarged, spreading to the neck and developing into nodules, plaque, partly purulent lesions, and sometimes fistulas associated with moderate pain and scarring. The patient reported no history of trauma or surgical procedure and could not recall any potential inducement of the lesions or previous receipt of immunosuppressant therapy.

**Figure:**
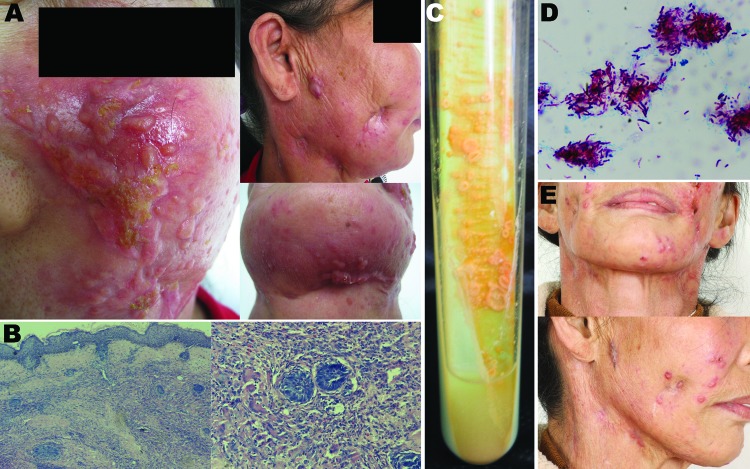
Cutaneous *Mycobacterium shigaense* infection in a 56-year-old immunocompetent woman, China. A) Plaques, scars with scabbing, nodules, and concave scars on the face and neck and papules and scarring on the submaxilla. B) Histopathologic results, showing hyperplastic epidermis and infiltration with lymphocytes, neutrophilic leukocytes, multinuclear giant cells, and epithelioid cells in the dermis. C) Samples streaked on Löwenstein–Jensen medium at 32°C formed smooth, yolk yellow creamy colonies. D) Ziehl–Neelsen staining of bacilli from the colonies that tested positive. E) Visible improvement of lesions after 4 months of treatment.

On physical examination, the patient was thin, with normal vital signs. Results of routine laboratory tests were unremarkable. Cephalic and cervical radiographs and computed tomography scans revealed lymphadenectasis of the neck only; a purified protein derivative test showed an erythema ≈8 mm in diameter on the forearm. Results of testing for HIV and human T-lymphotropic virus 1 antibody detection tests were negative. Cell-mediated immunity levels were detected by flow cytometry of peripheral blood cells; cell counts for CD3+, CD4+, CD8+ T-cells, and T-cell receptors and for CD19+ B-cells were within reference ranges.

Skin samples were collected from the face and neck of the patient and subjected to histopathologic examination, smear testing, and culture. Histopathologic examination showed a hyperplastic epidermis and noncaseating granulomatous infiltrates of lymphocytes, histiocytes, and multinucleate giant cells in the dermis (Figure, panel B). Results of Ziehl-Neelsen staining of smears for acid-fast bacilli and periodic acid–Schiff staining for fungi were negative; fungal and other bacterial cultures were sterile. Samples streaked on Löwenstein-Jensen medium at 32°C and 37°C for 4 weeks formed smooth, creamy, yolk yellow colonies (Figure, panel C); however, such colonies did not grow at 25°C and 45°C. Ziehl-Neelsen staining of samples from the colonies revealed acid-fast bacilli (Figure, panel D). Infection with a mycobacterium was suspected on the basis of these results.

The isolated bacilli were subjected to PCR restriction fragment-length polymorphism analysis and sequencing of the mycobacterial *hsp65* gene to identify the bacteria and strain (http://app.chuv.ch/prasite/index.html) (*2*,*3*). However, a match for the restriction pattern or the sequence of the isolated organism and *Mycobacterium* spp. was not found. Therefore, complete DNA sequences of the 16S rRNA, *hsp65*, and *rpoB* genes and the 16S–23S rRNA internal transcribed spacer (ITS) region were determined by using primers and PCR protocols described previously (*4*–*7*). The sequences of 16S rRNA, *rpoB* and the 16S–23S rRNA ITS region were identical to those of the nontuberculous mycobacterium species *M. shigaense*; the *hsp65* gene showed 94% similarity (Technical Appendix). 

Results of biochemical tests of the isolate were positive for *Mycobacterium* spp. by a 2-week culture on MacConkey agar and a heat catalase test but weakly positive by 3-day arylsulfatase and 2-week catalase testing (*8*). Results of nitrate reduction, semiquantitative catalase, growth in 5% NaCl medium, urease, and Tween 80 hydrolysis testing were negative. In vitro drug susceptibility was investigated by using the microdilution method, according to Clinical and Laboratory Standards Institute guidelines (*9*). The isolate was susceptible to moxifloxacin, amikacin, clarithromycin, rifampin, ethambutol, streptomycin, and ofloxacin.

The patient was treated with orally administered rifampin (450 mg 1×/d), moxifloxacin (400 mg 1×/d), and clarithromycin (500 mg 2×/d) for 6 months. The lesions subsided, leaving hyperplastic and atrophic scars (Figure, panel E). New nodules did not recur, and no notable side effects were found.

In general, culture-based identification methods using biochemical tests are slow and inadequate in differentiating species of mycobacteria. Laboratory methods with better performance, such as genetic investigations using nucleic acid amplification and sequencing, are increasingly used for identification. In this case, the complete gene sequences of 16S rRNA, 16S–23S rRNA ITS region, *rpoB,* and *hsp65* of the isolate were used to find the most consistent and highest scoring match across all 4 loci in GenBank (www.ncbi.nlm.nih.gov/genbank). 

In conclusion, our results strongly suggest that this chronic cutaneous infection in an immunocompetent patient was caused by *M. shigaen*s*e*. Our observations provide further evidence that this species should be classified as a nontuberculous mycobaterium that can cause disease in immunocompromised and immunocompetent patients. The isolate we identified was classified as clinically pathogenic and not an environmentally contaminating strain because it was isolated from multiple lesions and at different times. The lesions improved after treatment with clarithromycin and moxifloxacin, and the bacterium was not detectable thereafter.

Technical Appendix DNA sequences for 16S rRNA, 16S–23S rRNA internal transcribed spacer region, rpoB, and hsp65 genes of *Mycobacterium shigaense*.
