# Supplementary material for: Cutaneous Mycobacterium shigaense Infection in Immunocompetent Woman, China
Source: Emerg Infect Dis. 2013 May;19(5):819–20. doi: 10.3201/eid1905.121022 (PMC3647498; doi:10.3201/eid1905.121022)
Supplement: Technical Appendix — DNA sequences for 16S rRNA, 16S–23S rRNA internal transcribed spacer region, rpoB, and hsp65 genes of Mycobacterium shigaense. [file 12-1022-Techapp-s1.pdf]

Article DOI: http://dx.doi.org/10.3201/eid1905.121022

Cutaneous *Mycobacterium shigaense* Infection in Immunocompetent Woman, China

Technical Appendix

DNA sequences for *Mycobacterium shigaense* genes

All sequences are shown in a 5′→3′ orientation.

16S rRNA (*rrs*) (1,346 bp)

CGTGGGTAATCTGCCCTGCACTTCGGGATAAGCCTGGGAAACTGGGTCTAATACCGG

ATAGGACCACTTAGCGCATGCTTTGTGGTGGAAAGCTTTTGCGGTGTGGGATGGGCC

CGCGGCCTATCAGCTTGTTGGTGGGGTGACGGCCTACCAAGGCGACGACGGGTAGC

CGGCCTGAGAGGGTGTCCGGCCACACTGGGACTGAGATACGGCCCAGACTCCTACG

GGAGGCAGCAGTGGGGAATATTGCACAATGGGCGCAAGCCTGATGCAGCGACGCCG

CGTGGGGGATGACGGCCTTCGGGTTGTAAACCTCTTTCAGCAGGGACGAAGCGCAA

GTGACGGTACCTGCAGAAGAAGCACCGGCCAACTACGTGCCAGCAGCCGCGGTAAT

ACGTAGGGTGCGAGCGTTGTCCGGAATTACTGGGCGTAAAGAGCTCGTAGGTGGTTT

GTCGCGTTGTTCGTGAAAACCGGGGGCTTAACCCTCGGCGTGCGGGCGATACGGGC

AGACTGGAGTACTGCAGGGGAGACTGGAATTCCTGGTGTAGCGGTGGAATGCGCAG

ATATCAGGAGGAACACCGGTGGCGAAGGCGGGTCTCTGGGCAGTAACTGACGCTGA

GGAGCGAAAGCGTGGGGAGCGAACAGGATTAGATACCCTGGTAGTCCACGCCGTAA

ACGGTGGGTACTAGGTGTGGGTTTCCTTCCTTGGAATCCGTGCCGTAGCTAACGCAT

TAAGTACCCCGCCTGGGGAGTACGGCCGCAAGGCTAAAACTCAAAGGAATTGACGG

GGGCCCGCACAAGCGGCGGAGCATGTGGATTAATTCGATGCAACGCGAAGAACCTT

ACCTGGGTTTGACATGCACAGGACGCCGGCAGAGATGTCGGTTCCCTTGTGGCCTGT

GTGCAGGTGGTGCATGGCTGTCGTCAGCTCGTGTCGTGAGATGTTGGGTTAAGTCCC
GCAACGAGCGCAACCCTTGTCTCATGTTGCCAGCGCGTAATGGCGGGGACTCGTGA
GAGACTGCCGGGGTCAACTCGGAGGAAGGTGGGGATGACGTCAAGTCATCATGCCC
CTTATGTCCAGGGCTTCACACATGCTACAATGGCCGGTACAAAGGGCTGCGATGCCG
CAAGGTTAAGCGAATCCTTTTAAAGCCGGTCTCAGTTCGGATCGGGGTCTGCAACTC
GACCCCGTGAAGTCGGAGTCGCTAGTAATCGCAGATCAGCAACGCTGCGGTGAATA
CGTTCCCGGGCCTTGTACACACCGCCCGTCACGTCATGAAAGTCGGTAACACCCGAA
GCCAGTGGCCTAACCTTTTGGAGGGAGCTGTCGAAGGTGGGATCGGCGA
GGAAAGGCCCCTTCGGGGGTACT

16S–23S rRNA internal transcribed spacer region (197 bp)

TCTCCAATTGGTGGGGTGTGAGCCGTGAGGGGTTCTCGTCTGTAGTGGACGAGGGCC
GGGTGCACAACAGCAGGCAAATCGCCAGACACACTATTGGGCCCTGAGGCAACACT
CAGTTCTTCCGAGTGCTGTCCCTCCATCTTGGTGGTGGGGTGTGGTGTTTGAGTATTG
GATAGTGGTTGCGAGCATCTAAATGA

***rpoB* (3,362 bp)**

GCACCTAACCGAGTTTCCTTTGCCAAGCTTCGCGAACCGCTTGAGGTTCCGGGGCTT
CTCGATGTGCAGACCGACTCGTTTGAGTGGCTGATCGGCTCGCCCCGCTGGCGCGAG
AGCGCGACCGCACGCGGCGAGGTCAGCCCGGTGGGTGGCCTCGAAGAGGTGCTCTA
CGAGCTGTCGCCGATCGAGGACTTCTCCGGCTCGATGTCGCTGTCGTTCTCCGACCC
GCGTTTCGACGAGGTCAAGGCGCCGGTCGACGAGTGCAAAGACAAGGACATGACGT
ACGCAGCCCCGCTGTTCGTCACGGCCGAGTTCATCAACAACAACACCGGCGAGATC
AAAAGCCAGACGGTGTTCATGGGCGACTTCCCGATGATGACCGAGAAGGGCACCTT
CATCATCAACGGGACCGAGCGCGTGGTCGTCAGCCAGCTGGTCCGGTCGCCCGGTG
TGTACTTCGACGAGTCCATCGACAAGTCGACCGAGAAACTGCTGCACAGCGTGAAG
GTGATCCCGAGCCGGGGCGCCTGGCTGGAGTTCGACGTCGACAAGCGCGACACCGT
CGGCGTGCGCATCGACCGCAAGCGCCGGCAGCCGGTCACCGTGCTGCTCAAGGCGC
TGGGCTGGACCAACGAGCAGATCACCGAGCGGTTCGGCTTCTCCGAGATCATGATCT

CGACGCTGGAGAAGGACAATACCGCCGGCACCGACGAGGCGTTGCTGGACATCTAC

CGCAAGCTGCGACCGGGCGAGCCGCCGACCAAGGAATCCGCGCAGACCCTGCTGGA

GAACCTGTTCTTCAAGGAGAAGCGCTACGACCTGGCCCGGGTGGGCCGCTACAAGG

TCAACAAGAAGCTCGGCCTGCACGCGGGCGAGCCGATCACCTCGTCGACGCTGACC

GAGGAAGACGTCGTCGCCACCATCGAGTACCTGGTGCGGCTGCACGAGGGCCAGGC

CACGATGACGGTGCCCGGCGGCTCCGAGGTTCCGGTGGAGACCGACGACATCGACC

ACTTCGGCAACCGCCGGTTGCGCACGGTCGGTGAGCTGATCCAGAACCAGATCCGG

GTCGGTATGTCCCGGATGGAGCGCGTGGTCCGGGAGCGGATGACCACCCAGGACGT

CGAGGCCATCACGCCGCAGACCCTGATCAACATCCGCCCGGTGGTCGCCGCGATCA

AGGAGTTCTTCGGCACGAGCCAGCTGTCGCAGTTCATGGACCAGAACAACCCGCTG

TCGGGTCTGACCCACAAGCGGCGTCTGTCGGCGCTGGGCCCGGGTGGTCTGTCCCGT

GAGCGCGCCGGCCTGGAGGTCCGCGACGTGCATCCGTCGCACTACGGCCGGATGTG

TCCGATCGAGACCCCGGAGGGTCCGAACATCGGTTTGATCGGTTCGCTGTCGGTGTA

CGCGCGGGTGAACCCGTTCGGGTTCATCGAGACGCCGTACCGCAAGGTGGTCGACG

GAGTCGTCACTGACGAGATCCACTACCTGACCGCCGACGAGGAGGACCGCCACGTC

GTGGCGCAGGCCAACTCGCCGATCGACGCGGCGGGCCGGTTCGAAGAGGTGCGTGT

CCTGGTCCGCCGGAAGGCGGGCGAGGTCGAGTACGTGGCATCGTCCGAGGTGGACT

ACATGGACGTGTCGCCGCGCCAGATGGTGTCGGTGGCCACGGCCATGATCCCGTTCC

TCGAGCACGACGACGCCAACCGTGCCCTGATGGGTGCGAACATGCAGCGCCAGGCG

GTTCCGCTGGTGCGCAGCGAGGCGCCGCTGGTCGGCACCGGGATGGAGCTGCGCGC

CGCGATCGACGCCGGCGACGTCGTCGTCGCCGACAAGGCCGGGGTGATCGAGGAGG

TCTCCGCCGACTACATCACCGTGATGGCCGACGACGGCAGCCGGCACACCTACCGG

ATGCGCAAGTTCGCGCGGTCCAACCACGGCACCTGCGCCAACCAGTCGCCGATCGT

GGACGCCGGCGAACGCGTCGAAGCCGGCCAGGTGATTGCCGACGGCCCGTGCACCG

AAAACGGTGAGATGGCGCTGGGCAAGAACCTGCTGGTCGCGGTCATGCCGTGGGAG

GGGCACAACTACGAGGACGCGATCATCCTGTCCAACCGCCTGGTTGAAGAGGACGT

GCTCACCTCGATCCACATCGAGGAGCACGAAATCGACGCCCGCGACACCAAGCTGG

GCGCCGAGGAGATCACGCGGGACATCCCGAACGTCTCCGACGAGGTGCTCGCCGAC

CTGGACGAGCGCGGCATCGTGCGTATCGGTGCCGAGGTCCGCGACGGCGACATCCT

GGTCGGCAAGGTCACCCCGAAGGGTGAGACCGAGCTGACACCGGAGGAGCGGCTG

CTGCGCGCGATCTTCGGTGAAAAGGCCCGCGAGGTCCGCGACACGTCGCTGAAGGT

GCCGCACGGTGAGTCCGGCAAGGTGATCGGTATCCGGGTGTTCTCCCGCGAAGACG

ACGACGAACTGCCCGCCGGCGTCAACGAGCTGGTCCGGGTCTACGTCGCCCAGAAG

CGCAAGATCTCCGATGGCGACAAGCTGGCCGGACGGCACGGCAACAAGGGCGTCAT

CGGCAAGATCCTGCCGGTGGAGGACATGCCCTTCATGCCGGACGGCACTCCGGTCG

ACATCATCCTGAACACCCACGGTGTGCCGCGACGGATGAACATCGGTCAGATCCTG

GAGACGCACCTGGGGTGGGTCGCCAAGGCCGGCTGGAACATCGAGGGCACGCCCGA

CTGGGCGGCGAATCTGCCGGAGGACCTGCGCCACGCTGCGCCGGACCAGACGGTGT

CGACCCCGGTGTTCGACGGCGCCAAGGAGGAGGAGCTGCAGGGCCTGTTGTCCTGC

ACACTGCCTAACCGCGACGGCGACGTGATGGTCAACGCCGACGGCAAGGCGCAGCT

CTTCGATGGCCGCAGCGGGGAGCCGTTCCCGTACCCGGTGACCGTTGGCTACATGTA

CATCATGAAGCTGCACCACCTGGTGGACGACAAGATCCACGCCCGTTCCACCGGCC

CGTACTCGATGATCACCCAGCAGCCGTTGGGTGGTAAGGCGCAGTTCGGTGGCCAG

CGGTTCGGCGAGATGGAGTGCTGGGCCATGCAGGCCTACGGCGCCGCGTACACGCT

GCAGGAACTACTGACCATCAAGTCCGACGACACCGTCGGCCGGGTCAAGGTGTACG

AGGCGATCGTCAAGGGCGAGAACATCCCAGAGCCGGGGATTCCCGAGTCGTTCAAG

GTGTTGCTCAAGGAGCTGCAGTCGCTGTGCCTCAACGTCGAGGTGCTGTCGAGCGAC

GGTGCGGCGATCGAGCTGCGCGAGGGYGAGGACGAAGACCTCGAGCGCGC

***hsp65* (1,526 bp)**

GCTCATGCCCTTGCCGACGCGGTAAAGGTGACGTTGGGGCCCAAGGGTCGCAACGT

CGTCCTGGAGAAGAAGTGGGGCGCTCCCACGATCACCAACGATGGTGTGTCCATCG

CCAAGGAGATCGAGCTGGAGGACCCCTACGAGAAGATCGGCGCCGAGCTGGTCAAG

GAAGTCGCCAAGAAGACCGACGACGTTGCCGGTGACGGCACGACGACGGCCACCGT

GCTCGCTCAGGCACTCGTCAAAGAGGGCCTGCGCAACGTGGCGGCCGGCGCCAACC

CGTTGGCCCTCAAGCGCGGCATCGAGAAGGCTGTCGAGAGCGTCACCGAGACGCTG

CTCAAGTCGGCCAAGGACGTCGAGACCAAGGAGCAGATCGCGGCCACCGCGGGTAT

CTCCGCGGGCGACCAGTCGATCGGCGATCTGATCGCCGAGGCCTTGGACAAGGTCG

GCAACGAGGGCGTCATCACTGTCGAGGAGTCCAACACCTTCGGCCTGCAGCTCGAG

CTCACCGAGGGCATGCGGTTCGACAAGGGCTACATCTCGGGCTACTTCGTCACCGAC

CCCGAGCGTCAGGAAGCGGTCCTCGAGGACCCCTACATCCTGCTGGTCAGCTCCAA

GGTGTCGACCGTCAAGGATCTGCTGCCCCTGCTGGAGAAGGTCATCCAGTCCGGCA

AGCCGCTGCTGATCATCGCCGAGGACGTCGAGGGCGAGGCGCTGAGCACCCTGGTC

GTCAACAAGATCCGCGGCACGTTCAAGTCGGTGGCGGTCAAGGCCCCCGGTTTCGG

TGACCGTCGCAAGGCGATCTTGCAGGACATCGCCATCCTGACCGGCGGCCAGGTGA

TCAGCGAAGAGGTCGGCCTGTCGTTGGAGAACACGGACCTTTCGCTGCTGGGCAAG

GCCCGCAAGGTCGTGATCACCAAGGACGAGACCACCGTCGTCGAGGGCGCCGGTGA

CACCGACGCCATCGCCGGCCGGGTGGCCCAGATCCGCGCCGAGATCGAGAACAGCG

ACTCCGACTACGACCGCGAGAAGCTGCAGGAGCGTCTGGCCAAACTGGCCGGCGGT

GTTGCGGTGATCAAGGCCGGCGCCGCCACCGAGGTGGAGCTCAAGGAGCGCAAGCA

CCGCATCGAGGACGCGGTGCGCAACGCCAAGGCCGCCGTCGAGGAGGGCATCGTCG

CCGGTGGTGGCGTGGCCCTGCTGCAGGCGGCCCCGTCGCTGGACAAGCTGAAGCTG

TCGGGTGACGAGGCGACCGGCGCCAACATCGTCCGCGTGGCGCTGTCGGCTCCGCT

GAAGCAGATCGCCTTCAACTCCGGGCTGGAGCCCGGCGTGGTCGCCGAGAAGGTCC

AGAACTCGCCCGCGGGTACCGGTCTGAACGCCGCCACCAACGAGTACGAGGACCTG

CTCAAGGCCGGCGTTGCCGACCCGGTCAAGGTGACCCGTTCGGCGCTGCAGAACGC

GGCGTCCATTGCGGGCCTGTTCCTGACGACCGAGGCCGTCGTTGCCGACAAGCCGG

AGAAGGCCGCCGC
